# Imagine all you want, but…

**DOI:** 10.5195/jmla.2022.1415

**Published:** 2022-07-01

**Authors:** Taran Ley

**Affiliations:** 1 tley65@siumed.edu, Medical Library Director, Southern Illinois University School of Medicine - Medical Library, Springfield, IL.

**Keywords:** 3D printing, technology, medical models, patient education, medical curriculum

## Abstract

3D printing is an emerging trend in medical care [1]. Medical libraries can play a key role in advancing this new technology [2]. Using a National Library of Medicine (NLM) grant, the medical library was able to purchase a basic 3D printer to create models for patient care and medical education. Despite a slow rollout for the new technology, there was a strong need once word of mouth spread about the new 3D printer. The one-year grant cycle, as well as the following three years, provide supporting evidence that even a basic 3D printer can advance patient care for clinicians and improve medical education for students [3]. The popularity of the technology, clinical support and demand, as well as student interest can drive the program forward on its own and support the medical library's mission to improve community care and create an environment of enhanced learning [1].

## BACKGROUND

When you are a new director, your objective, whether you admit it or not, is to impress. You want to get everything right and set the world on fire, or at least gain notice of the C-Suite at your institution. You tell yourself it's to make the library better, advance the mission, strategically plan for the future…..and on it goes. But I believe the best leaders are the ones who say, “That's a new concept I know nothing about! Tell me more.” I found myself saying those humble words to my technology coordinator during my first month as a medical library director. She was describing 3D printing to me and how she had used a borrowed printer to make a model of a child's spinal column for one of our orthopedic surgeons. I kept thinking “How does a copier make a spinal column?” I realize of course, everyone knows what 3D printing is, but in January 2017, I had no idea what she was talking about. I was far from impressing her or anyone in the C-Suite at that point, but that was okay. Once it was on my radar, I was all in. Being more creative than pragmatic at that moment, I immediately envisioned a space where medical students, educators, researchers, and clinicians could create patient models for education and surgical planning. We would create an “Imagineering Lab” with a state-ofthe-art 3D printer that would rival Mayo Clinic itself [4]. It was going to be fantastic!

It was about that time reality set in. I was told, “Imagine all you want, but the medical library does not:

- have the funds to purchase a printer,
- have the space for a printer, and
- have the clinical expertise to print 3D medical models.”

A wise person once said, “Nothing worth doing is ever easy” [5]. I held on to that for the next four years.

## CASE PRESENTATION

### Purchasing a 3D Printer

We had no money to purchase even a basic 3D printer, let alone one capable of producing medical models. Funding challenges like this are familiar to every library. Library directors lay awake at night wondering what they will need to cut next just to make ends meet, let alone fund a new program. About the time we were discussing this problem, the National Library of Medicine–Greater Midwestern Region (NLM-GMR) sent out a call for grant proposals. Our library is both an Outreach Library and Resource Library for the NLM-GMR (now Region 6). Reaching out to staff at the NLM's regional office in October 2017, I pitched a proposal to purchase a 3D printer through their Health Information Outreach Award (up to $50,000). Their response was promising, so I began working out a formal application for submission in April 2018. My technology coordinator and I sat in my office looking at the Makerbot website and trying to determine how much filament we would need to produce various models. Like other libraries, we felt the Makerbot was a good choice [6]. By the time I had completed the grant, we were using our best guesses on printer size and capability, our ability to understand the machine, supplies we would need for setup, and the amount of filament to last our first year, along with the ever-important service contract for when we broke the printer. We were both terrified and thrilled when we were awarded the grant on April 26, 2018, for $17,293 [7] and purchased the following:

- Makerbot Replicator Z18 Large 3D Printer,
- assorted filament in a variety of colors,
- cart,
- build plates,
- extruder, and
- a one-year service contract.

We were off and running!

## SPACE—IF YOU BUILD IT, THEY WILL COME.

We now had a printer, but nowhere to put it. It was a good problem to have, but a problem nonetheless. Everyone knows space is a rare commodity in a library. If an external party isn't trying to land grab, you have a host of internal power struggles with collection growth, community need, study space, etc. The most logical location would be existing space that was underutilized. My attention focused on a special collections room we had. It was in a prime location in our faculty office hallway, behind the circulation desk. The room had never been appropriately retrofitted for a special collection, and we continuously battled heat and humidity in the space. In June 2018, we decided moving the books out of the special collection room and to our technical services area would provide space for an Imagineering Lab. Still not ideal for the collection, but better than its present location. The move opened up a room that measured roughly 18 by 18 feet and would comply with our grant requirement that the printer stay locked up or under direct supervision at all times. The printer was currently housed in the technology coordinator's office which was small and cramped. The proposed space had already been part of a major renovation in 2015, so it had new paint, carpet, and a raised floor that provided updated wiring and outlet access. In mid-2018 we began moving the technology coordinator, and the Makerbot, out of the office and into the new space. Our momentum continued!

### Expertise and Support

Under the terms of our grant, our plan was to reach out to the directors of our health care clinics to promote the 3D printer and work with them to develop models for:

- Anesthesiology
- Family & Community Medicine
- Neurology
- Obstetrics & Gynecology
- Pathology
- Pediatrics
- Psychiatry
- Radiology
- Surgery

We hoped to garner anatomical expertise from the doctors and support from their departments. The first meeting, in June 2018, was a disappointment. The Anesthesiology Department had no real need for a 3D printer but thanked us for the offer. Next was Family and Community Medicine. The department was polite but had no real need either, and a meeting with the director never materialized. As we began discussing our next steps, requests for models suddenly began pouring in! Word-of-mouth had spread about the library's new printer! Taking a page from the private sector, you can market and advertise all you want, but it is hard to beat word-of-mouth referrals. I soon realized that our early work with the borrowed printer had already convinced some clinicians that 3D printing had tremendous value. Their use of the simple models from the borrowed printer had a ripple effect among the departments. Our first request was from the Department of Otolaryngology Head and Neck Surgery, which requested a skull model from a CT scan.

**Image 1:**
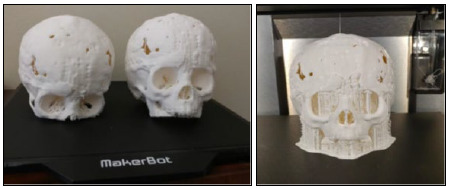
Child's skull. The patient had craniosynostosis and had prior surgery to reconstruct the skull. The patient still had defects (visible on the model), and the skull served as a road map in the operating room to repair the defects. It was also a useful teaching tool for talking with the family [personal communication from Dr. M. Johnson to T. Ley, October 15, 2021].

**Image 2:**
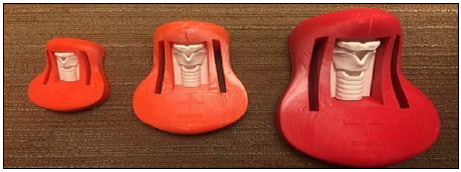
Cricothyroidotomy trainers (infant, child, and adult). Printed for the surgical skills lab.

The surgical skills team [8] soon heard about the 3D printer as well. The team was so excited they came to the medical library en masse wearing their scrubs. As they circled the 3D printer, they were full of questions and excited ideas of what they would like us to print. Their first request was for training models (see Image 2).

As interest in the printer surged, two doctors emerged who understood the impact 3D printing could have on health care. The first is a facial plastic and reconstructive surgeon in the Department of Otolaryngology, Head and Neck Surgery. He is a perfect example of how 3D printing is being used for surgical simulation and preoperative planning [9].

“Previously, in preparation for surgery, we would review imaging. This can be challenging and at times, abstract to visualize. With a 3D model, we have direct assessment and appreciation of anatomy. We use virtual surgical planning to prepare plates, implants, location of osteotomies [procedures altering bones], and anticipate the steps of surgery” [10]. We have used models to assess where we will make our osteotomies. I have used tools to simulate the bone cuts and see how the shape can be manipulated or move….they have been useful road maps during surgery to locate anatomy or show/explain/teach patients in clinic preoperatively” [personal communication from Dr. M. Johnson to T. Ley, October 15, 2021].

The second doctor is an assistant professor and clinical radiologist who became the medical library's strongest supporter and staunchest ally throughout our journey. Like other radiologists, he recognized the value of 3D printing in a medical environment early on [11] and began advocating for a 3D printer in hospitals. Unlike me, everyone knew what a 3D printer did, but no one was willing to commit funds for a purchase. When he heard about the medical library's 3D printer from the surgical skills team [8], he began making frequent visits to meet with our technology coordinator and discuss models. His daily work with clinicians gave the medical library a solid bridge between the school and the clinics. He showed models we had produced and acted as an intermediary for requests from other clinicians. It was the perfect collaboration. Early models were printed using the National Institute of Health's (NIH) 3D Print Exchange, Embodi 3D and Thingiverse [12]. We soon began translating patient CT scans to .stl files for printing. Two heart models for a cardiothoracic surgeon produced the following comments:

“Doctor loved it. Said it made a big difference in how he handled the case and the patient would have probably had complications if not for what he saw on the model! He wants to do a write-up of the case and promote the technology!” [text message from Dr. R. Hidalgo to K. WhiteApril 15, 2021].

From 2018-2021, the medical library produced approximately 70 3D printed models for medical education, 38 for clinical care, and 40 for display or to give to visitors.

Clinical care models for presurgical planning, patient education, and research devices include a(n) heart, kidney, lungs, skull, palate with cleft, aorta, trachea, vertebrae, pelvis, ribcage, and femur. Typically, a model will show the fracture or tumor in order to advance patient care and education. All models have been printed at no charge.

We have been amazed to see how even a basic 3D model can assist in surgical planning. We were even more amazed when the local hospital reached out to us, during COVID-19 (2020), to print personal protective equipment (PPE). Although we did not have the capability to print CDC-compliant PPE, the request clearly indicated administrators within the health care community knew about the library's printer.

The 3D printer personifies the medical library's mission to advance and support the education of our faculty, students, and residents, to improve the quality of care for our local residents, and to provide an environment of learning and scholarship that helps advance the forefront of medical knowledge and technology [13]. Like other institutions, “the library has established itself as a technology hub, or central place, for collaboration to meet the challenging needs of the university's health sciences curricula” [2].

## DISCUSSION

It was impossible to predict the popularity and support of the medical library's 3D printer back in 2017. Although I imagined great things, bringing the printer to fruition has been a daunting task. We faced the usual setbacks expected with any new program and this technology. Some models do not look impressive in only one color, particularly when trying to show a tumor next to an organ. Delicate models can be difficult to clean and may break easily. Moving the machine, even slightly, can disturb the sensitive leveling calibrations. That being said, the Makerbot has proven to be a reliable little workhorse that continues to impress us. Without the support of our clinicians and the vision of our technology coordinator, success would have been elusive. The Imagineering Lab has become so popular, radiology now teaches 3D printing as part of the third-year medical students' clinical rotations. Studies have already proven that “students using 3D printed models obtained higher results in tests checking their anatomical knowledge” [14]. A formal elective is being planned with the Department of Medical Education and Curriculum to improve the student experience. The medical library's technology coordinator will be the instructor for the course. We are currently in discussion to purchase Stratasys' new J5 Medijet printer. The J5 can print with multi-materials and colors. Multiple models can also be printed in a single print. With the additional purchase of a waterjet system, models will be cleaned and smoothed to a more professional polish and look. The cycle continues as we discuss funding and space yet again, but the previous four years have helped us build a coalition of supporters and a network of specialists to assist in that process. Like other institutions that have initiated 3D printing programs, the library's visibility has improved, traffic has increased, and we have developed relationships that had not previously existed [6]. If I were asked what has been the greatest lesson learned throughout the process, I would have to say two things:

- Dream big—even if you fall short, you have still accomplished something great! Despite having limited funding, space, and expertise, we were able to create space for a new education model and patient service.
- Share your dream with others—you will accomplish so much more with those who believe in you and your vision! Throughout the process, the enthusiasm of the library staff and support of our doctors kept us moving forward.

## Data Availability

There are no data associated with this article.
